# Modulation of the Host Immune Response by Schistosome Egg-Secreted Proteins Is a Critical Avenue of Host–Parasite Communication

**DOI:** 10.3390/pathogens10070863

**Published:** 2021-07-08

**Authors:** Jack P. Carson, Geoffrey N. Gobert

**Affiliations:** School of Biological Sciences, Queen’s University Belfast, 19 Chlorine Gardens, Belfast BT9 5DL, UK; jcarson22@qub.ac.uk

**Keywords:** *Schistosoma mansoni*, *Schistosoma japonicum*, *Schistosoma haematobium*, schistosome, egg, excretory/secretory

## Abstract

During a schistosome infection, the interactions that occur between the mammalian host and the parasite change rapidly once egg laying begins. Both juvenile and adult schistosomes adapt to indefinitely avoid the host immune system. In contrast, the survival of eggs relies on quickly traversing from the host. Following the commencement of egg laying, the host immune response undergoes a shift from a type 1 helper (Th1) inflammatory response to a type 2 helper (Th2) granulomatous response. This change is driven by immunomodulatory proteins within the egg excretory/secretory products (ESPs), which interact with host cells and alter their behaviour to promote egg translocation. However, in parallel, these ESPs also provoke the development of chronic schistosomiasis pathology. Recent studies using high-throughput proteomics have begun to characterise the components of schistosome egg ESPs, particularly those of *Schistosoma mansoni*, *S. japonicum* and *S. haematobium*. Future application of this knowledge may lead to the identification of proteins with novel immunomodulatory activity or pathological importance. However, efforts in this area are limited by a lack of *in situ* or *in vivo* functional characterisation of these proteins. This review will highlight the current knowledge of the content and demonstrated functions of schistosome egg ESPs.

## 1. Schistosomiasis

Schistosomiasis affects over 200 million people worldwide. The disease is endemic primarily in the economically developing regions of Asia, South America and sub-Saharan Africa, and results in approximately 200,000 deaths annually [1,2]. *Schistosoma mansoni* (Africa, South America and the Middle East), *S. japonicum* (Southeast Asia) and *S. haematobium* (Africa) are the three schistosome species mainly responsible for human disease [1]. Africa carries the greatest burden of schistosomiasis, accounting for over 90% of global cases, due to the overlapping habitats of both *S. mansoni* and *S. haematobium* [3,4]. The highest level of schistosomiasis is reported in Nigeria (29 million cases), which is closely followed by the United Republic of Tanzania (19 million cases) and the Democratic Republic of Congo (15 million cases). *Schistosoma japonicum* is endemic in regions of China, the Philippines, and limited areas of Indonesia. In China it is estimated that less than 100,000 people are currently infected, whereas, in the Philippines, 6.7 million people are thought to live in endemic regions and 1.8 million of these are believed to be at direct risk of infection. Elimination of schistosomiasis in multiple provinces in China has recently been reported [5].

Human infection occurs when water-borne cercariae penetrate the host epidermis and transform into schistosomula. The juvenile parasites then circulate via dermal blood vessels and migrate to the intestinal mesenteric veins (*S. mansoni* and *S. japonicum*) or the venous plexus of the bladder (*S. haematobium*) [6]. Schistosomula mature into sexually distinct adult worms during this migratory phase and, upon completing their migration, form mating pairs that produce eggs, which are subsequently shed either in the faeces or urine [6]. Eggs that fail to shed from the host are instead carried away in circulation and become deposited within host tissues, commonly the liver sinusoids (*S. mansoni* and *S. japonicum*) [7] or the mucosa of the bladder wall (*S. haematobium*) [8]. These trapped eggs are eventually destroyed within a granuloma derived from host immune cells, however, the breakdown of a granuloma after egg death leaves behind a fibrous plaque. The accumulation of these plaques throughout long-term infection leads to reduced liver function and the development of debilitating schistosomiasis pathologies.

The pathology caused by schistosomiasis parallels changes in the parasite’s lifecycle. Migrating schistosomula drive a moderate host Th1-mediated inflammatory immune response which changes to a stronger Th2-mediated immune response against parasite eggs, many of which become trapped in host tissues [7]. This Th2 response develops alongside the beginning of egg laying and is brought about by protein components released from eggs within the ESPs. These proteins modulate the responses of host immune cells, altering their cytokine and chemokine expression profiles towards a Th2 phenotype [7]. This modulation is considered to be beneficial to the traversing egg in effectively exiting from the host; studies using immunocompromised mice unable to adopt this Th1-to-Th2 shift, and thus unable to form effective granuloma, present significantly diminished levels of egg excretion in *S. mansoni* and *S. japonicum* infections [9,10,11]. It is not fully understood how granuloma formation impacts egg migration; however, it may act to protect the egg from inflammatory damage as it migrates through the intestinal tissues and into the lumen [12]. Furthermore, blocking this immune shift and instead maintaining a dominant Th1 response throughout chronic schistosomiasis results in significant host mortality in mouse models [13]. This is thought to occur due to unmodulated egg-related immunopathology, causing excessive inflammatory tissue damage in response to mounting egg burden [13].

Given the conflicting roles egg-secreted proteins carry out in driving the pathology associated with chronic schistosomiasis while also promoting host survival, identifying which specific proteins within the egg ESPs carry out these activities *in vivo* will further our understanding of the dynamics formed between host and parasite during chronic disease. Furthermore, it will also allow for the identification of novel immunomodulatory proteins that may have pharmacological relevance for the treatment of other conditions.

## 2. Schistosome Egg Proteomics—Technologies and Key Studies

The first proteomic mass spectrometry analysis of the secretome of schistosome eggs was published in 2007 when Cass and colleagues described 188 proteins within *S. mansoni* egg ESPs [14]. Since this original report, mass spectrometry has been used in tandem with 1- and 2-dimensional gel electrophoresis (1DE and 2DE), as well as OFFGEL electrophoresis, to further characterise the egg secretomes of *S. mansoni*, *S. japonicum* and *S. haematobium*. The *S. japonicum* egg secretome, investigated using 1DE/mass spectrometry and found to contain 95 proteins, was recently reported [15], while the *S. haematobium* egg secretome was found to contain 138 proteins after investigation using OFFGEL and mass spectroscopy [16]. Our own group carried out a concurrent analysis of the egg secretomes of the three main species infecting humans (*S. mansoni*, *S. japonicum* and *S. haematobium*) [17]. Our approach was direct, using unseparated preparations in solution, and resulted in the identification of 266, 90 and 50 proteins within the ESP profiles, respectively (Supplementary Tables S1–S3) [17]. The differences between our data for *S. mansoni* and those of the original study [14] were anticipated, and we attribute them to the improved reference databases and search algorithms that have become available over the intervening decade. The variation in the number of proteins detected in *S. haematobium* egg ESPs between our own work and that of the 2019 study by Sotillo et al. [16] is potentially due to the technical differences between the two studies. Sotillo and colleagues [16] analysed *S. haematobium* crude ESP using OFFGEL electrophoresis, separating their preparations into 24 fractions which were then each subjected to mass spectroscopy analysis, contrasting with our method of using unfractionated protein preparations. The OFFGEL procedure is also considered to be more sensitive than other proteomics methods [18]. Other studies have employed similar methods to describe the secretomes of adult *S. mansoni* and *S. japonicum* worms [19,20].

### 2.1. Immunomodulatory S. mansoni Egg ESPs

A summary of the distribution and function of the proteins discussed below can be seen in Table 1. As the most common experimental model for schistosomiasis, *S. mansoni* presents the most egg ESPs that have functional characterisation. IPSE/α-1, omega-1 and major egg antigen P40 are three of the most prominent proteins among these egg ESPs [21,22]. The hepatotoxic glycoprotein IPSE/α-1 has been shown to bind interleukin (IL)-8, thus inhibiting the migration of neutrophils to the site of a granuloma, and to induce the degranulation of, and release of IL-4 and IL-13 from, basophils [23,24]. As well-known mediators of the Th2 response, IL-4 and IL-13 carry out essential roles in the initiation of fibrogenesis in the liver and other organs, including driving the production of collagen [25]. Omega-1, another hepatotoxic glycoprotein, possesses T2 ribonuclease activity. Omega-1 is known to be taken up by dendritic cells (DCs), wherein it utilises its ribonuclease activity to degrade cellular rRNA and mRNA (ribosomal and messenger RNA) and influence DC function. Following exposure to omega-1, DCs have been shown to express reduced levels of IL-12, a cytokine involved in promoting the development of the Th1 response [26]. In contrast to both of these proteins, P40 has demonstrated anti-fibrotic activity and is associated with regulatory roles. In mouse models, the stimulation of lymphocytes by P40 induces both interferon (IFN)-γ and IL-2 expression, both of which promote a Th1 response [27].

Other proteins within the egg ESPs that have a proposed modulatory impact on the host immune response include *Sm*PEPCK, *Sm*VAL9 and peroxiredoxin. In murine models, *Sm*PEPCK induces CD4+ T cell proliferation and primes them to release IFN-γ, IL-4 and IL-5, resulting in a mixed Th1-Th2 response [36,37]. *Sm*VAL9 has been shown in mouse studies to induce host extracellular matrix (ECM) remodelling through the alteration of MMP (matrix metalloproteinase) and TIMP (tissue inhibitors of metalloproteinase) enzyme expression. MMPs and TIMPs are central in the regulation of the formation and resolution of fibrogenesis associated with hepatic schistosomiasis [44]. This dysregulation by *Sm*VAL9 promotes host ECM degradation, which likely improves egg translocation through the host intestinal lumen [38]. Finally, peroxiredoxin primes a host Th2 immune response by promoting the alternative activation of macrophages (AAMs). These AAMs in turn drive the transcription of the pro-Th2 cytokines IL-4, IL-5 and IL-13 in naïve CD4+ cells [39].

### 2.2. Immunomodulatory S. japonicum Egg ESPs

*S. japonicum* egg ESPs contain several ribonucleases, including T2, Oy and X25, which share sequence identity with omega-1 in *S. mansoni* (31%, 52% and 35% respectively) and its own P40 variant (66% sequence identity with *S. mansoni*-P40). In murine studies, ribonuclease T2 stimulates a distinct Th2 immune response in the host by affecting the maturation of DCs, inducing the production of anti-inflammatory IL-10 by macrophages and the upregulation of IL-4 levels in the serum [40]. In contrast, *S. japonicum*-P40 inhibits fibrogenesis by reducing the activation of hepatic stellate cells and promoting apoptosis and senescence in these cells [33,34,35].

*Sj*E16.7 and *Sj*97 are two additional significant proteins found within the egg ESPs. *Sj*E16.7 is a strong neutrophil chemoattractant and inducer of inflammation [41], while *Sj*97 can inhibit components of the complement pathway and also bind directly to the Fc region of human immunoglobulins [42,43]. Sj97 has previously been associated with the cercarial and schistosomula stages in immunolocalisation studies [45]. *Sj*E16.7 likely functions in combination with *Sj*-P40 in order to regulate the developing Th2 immune response, whereas *Sj*97 is hypothesised to reduce complement-mediated cytotoxicity against juvenile and adult worms by interacting with complement-associated proteins [45]. A role of *Sj*97 in the egg stage is not clear, but it is possible that it has a similar protective function in reducing complement-mediated damage against eggs.

### 2.3. Immunomodulatory S. haematobium Egg ESPs

Limited research has been carried out to characterise the potential roles of *S. haematobium* egg-secreted proteins in the development of pathology or the modulation of the host immune response. *Schistosoma haematobium* eggs do, however, secrete an IPSE/α-1 ortholog [30] with 67% sequence identity to *Sm-*IPSE/α-1. *Schistosoma haematobium*-IPSE/α-1 is internalised by bladder cells in culture (HTB-9), where it localises to the nucleus, indicating a potential role in regulating host cell gene expression [30]. A major consequence of chronic schistosomiasis *haematobium* is the possible occurrence of urogenital squamous cell carcinoma [8,46,47]. The potential for *S. haematobium* egg-secreted proteins to act as carcinogens has not been investigated to any great extent. However, IPSE/α-1 is a major component of *S. haematobium* egg ESPs and is capable of inducing angiogenesis and cell proliferation [16,31] and, therefore, may be involved in carcinogenesis during urogenital schistosomiasis. While the functional characterisation of *S. haematobium* egg-secreted proteins is particularly lacking, comparisons can be made between these proteins and those in *S. mansoni* and *S. japonicum* egg ESPs. One example is the protein *Sh*E16, which shares high (70%) sequence identity with the *S. japonicum* egg ESP protein *Sj*E16, although it has not been shown if *Sh*E16 is also a chemoattractant for neutrophils in a similar manner to the *S. japonicum* protein.

## 3. Conclusions and Future Directions

During a schistosome infection, the commencement of egg laying marks a critical period of change to the host–parasite dynamic both in terms of the phenotype of the host immune response and the stimuli that drive pathology. While juvenile and adult schistosomes survive by avoiding the host immune response, eggs instead secrete proteins with immunomodulatory activities to manipulate the host response in a direction that promotes egg/host survival and egg transmission. Several recent studies have described the protein contents of *S. mansoni*, *S. japonicum* and *S. haematobium* egg ESPs; however, the function of many of these proteins remains undefined. Future research should consider characterising the *in vivo* role of these proteins, as doing so may lead to the identification of novel mediators of host–parasite interactions that are relevant during chronic schistosomiasis, or immunomodulatory proteins that could be repurposed as therapeutics for diseases characterised by host Th1 or Th2 responses.

The schistosome egg ESPs described here are immunomodulatory proteins that predominantly incite a host Th2 immune response and are thought to influence the Th1-to-Th2 shift that follows patency. However, few proteins that promote Th1 and mixed Th1/Th2 phenotypes have been identified, which complicate the impact that egg ESPs have on the host immune response. These proteins are expected to be involved in controlling the intensity of the developing the Th2 response that is associated with chronic schistosomiasis, giving the egg a level of control over the progression of the immune shift. As outlined here, much of the available data suggests that *S. mansoni* egg ESPs contain a greater variety of immunomodulatory components than those of *S. japonicum* or *S. haematobium*. The relatively fewer immunomodulatory proteins associated with *S. japonicum* egg secretions could represent a causative factor to the increased disease intensity this species causes in comparison to *S. mansoni* infections [1]. Generally, this increased pathology is credited to the greater fecundity of mature *S. japonicum* worm pairs, which results in a greater number of eggs trapped within the host tissues and an associated increase in hepatic inflammation and fibrosis [48]. However, the reduced diversity of immunomodulatory proteins within the *S. japonicum* egg ESPs could reflect an inability to effectively modulate the host immune system compared to *S. mansoni* eggs. This deficiency would result in a reduced Th1-to-Th2 shift and a more protracted chronic inflammatory state, which could produce greater pathology.

Many of the questions raised in this review could be answered by new disease models and technologies. One technology that could be used to study the functionality of specific proteins from schistosome eggs is an *ex vivo* model, known as the Precision Cut Liver Slice (PCLS) platform, using naïve mouse liver. PCLS replicates the cellular microenvironment of the *in vivo* liver and has been employed in metabolism and toxicity studies, as well as chemical and surgical fibrogenesis models [49]. The responses of mouse liver slices to *Sj*-SEA (*S. japonicum*-crude soluble egg antigen) using PCLS has been published [50], with tissue remaining unfixed, unfrozen, and alive throughout for subsequent *ex vivo* studies. The addition of *Sj*-SEA resulted in only minimal changes to overall hepatocellular morphology, and no significant increase in hepatotoxicity up to 48 h post-treatment. SEA concentrations were representative of those released by parasite eggs based on previous *in vivo* experimental infections and known liver egg burdens [51]. Going forward, this model could be used with egg ESP fractions, or individual proteins isolated from crude egg ESPs, to further clarify the role of these proteins in the *in vivo* setting.

## Figures and Tables

**Table 1:** The effect(s) of immunomodulatory proteins within schistosome egg ESPs.

Protein	Species	Effect(s)	Reference(s)
IPSE/α-1	*S. mansoni*	Induces cell death in hepatocytes	[28]
		Interacts with IL-8 to inhibit neutrophil chemotaxis	[23]
		Induces degranulation of basophils and subsequent release of IL-4 and IL-13	[29]
	*S. haematobium*	Localises to the nucleus of HTB-9 bladder cells	[30]
		Induces angiogenesis and proliferation in EOMA endothelial cells and HCV-29 urothelial cells respectively	[31]
Omega-1	*S. mansoni*	Induces cell death in hepatocytes	[32]
		Degrades dendritic cell RNA to reduce the release of IL-12	[26]
P40	*S. mansoni*	Induces murine lymphocytes to release IFN-γ and IL-2	[27]
	*S. japonicum*	Inhibits TGF-β1-induced activation in hepatic stellate cells	[33]
		Induces senescence and apoptosis in hepatic stellate cells	[34,35]
*Sm*PEPCK	*S. mansoni*	Induces proliferation and release of IFN-γ, IL-4 and IL-5 from murine CD4+ T cells	[36,37]
*Sm*VAL9	*S. mansoni*	Alters the expression profiles of MMP and TIMP enzymes in murine macrophages to promote ECM degradation	[38]
Peroxiredoxin	*S. mansoni*	Promotes the differentiation of alternatively activated macrophages	[39]
Ribonuclease T2	*S. japonicum*	Inhibits dendritic cell maturation, induces macrophages to release IL-10 and increases IL-4/decreases IFN-γ levels in the serum	[40]
*Sj*E16.7	*S. japonicum*	Promotes neutrophil chemotaxis	[41]
*Sj*97	*S. japonicum*	Interacts with complement-associated proteins C8 and C9 to inhibit complement	[42]
		Interacts with the Fc region of human antibodies	[43]

## Data Availability

Data sharing not applicable.

## Supplementary Materials

The following are available online at https://www.mdpi.com/article/10.3390/pathogens10070863/s1, Supplementary Table S1. Comparison of ESP data generated by Carson et al. [17] with reports from *S. mansoni* [14,19]. Gene Name, Accession No., Protein ID, Molecular Weight, GO ID, GO name and if the protein was reported previously in other studies, are provided. Supplementary Table S2. Comparison of ESP data generated by Carson et al. [17] with reports from *S. japonicum* [15,20]. Gene Name, Accession No., Protein ID, Molecular Weight, GO ID, GO name and if the protein was reported previously in other studies, are provided. Supplementary Table S3. Comparison of ESP data generated by Carson et al. [17] with reports from *S. haematobium* [16]. Gene Name, Accession No., Protein ID, Molecular Weight, GO ID, GO name and if the protein was reported previously in other studies, are provided.
